# Association of Arterial Stiffness Indices with Framingham Cardiovascular Disease Risk Score

**DOI:** 10.31083/j.rcm2308287

**Published:** 2022-08-16

**Authors:** Lin Jin, LanYue Tong, CuiQin Shen, LianFang Du, JianYing Mao, LiPing Liu, ZhaoJun Li

**Affiliations:** ^1^Department of Ultrasound, Jiading District Central Hospital Affiliated Shanghai University of Medicine & Health Sciences, 201800 Shanghai, China; ^2^Department of Ultrasound, First Hospital of Shanxi Medical University, 030001 Taiyuan, Shanxi, China; ^3^Department of Ultrasound, Shanghai General Hospital Jiading Branch, Shanghai Jiaotong University School of Medicine, 201812 Shanghai, China; ^4^Department of Ultrasound, Shanghai General Hospital, Shanghai Jiaotong University School of Medicine, 200080 Shanghai, China; ^5^Department of Ultrasound, Guanghua Hospital Affiliated to Shanghai University of Traditional Chinese Medicine, 200052 Shanghai, China

**Keywords:** arterial stiffness, arterial velocity pulse index, arterial pressure volume index, Framingham score

## Abstract

**Purpose:**

The new non-invasive arterial stiffness indices, arterial velocity pulse index (AVI) and arterial pressure volume index (API) are known to be associated with cardiovascular disease risk. The present study aimed to examine the “dose-response” associations between AVI, API and Framingham cardiovascular disease risk score (FCVRS).

**Methods:**

This survey included individuals with arterial stiffness indices collected at age 18 years and older. We used Pearson’s correlation coefficients and multivariate linear analyses to evaluate associations of AVI and API to other variables. The associations between FCVRS and AVI, API were analyzed by restrictive cubic spline.

**Results:**

4311 people were included in the full study population, including 2091 males and 2220 females. In restricted cubic spline regression models, AVI or API had significant U-shaped associations with FCVRS, with the lowest risk score of cardiovascular disease was 8 units or 18 units, respectively. After AVI increased to 12 units, FCVRS increased rapidly until AVI was 27 units, and the FCVRS increased relatively flat afterward. For API, results were similar. When API increased to 23 units, the FCVRS increased rapidly, and after API was 52 units, FCVRS increased relatively flat.

**Conclusions:**

AVI or API had U-shaped associations with FCVRS. The associations may provide a new perspective for early treatment or lifestyle modifications to prevent cardiovascular diseases.

## 1. Introduction

Cardiovascular disease (CVD) remains the leading cause of morbidity and mortality globally [1]. The course of CVD is generally long. It often takes years to decades from the occurrence of lesions to the development of malignant cardiovascular events (such as myocardial infarction and stroke). Therefore, it has become a consensus in the medical community to move the prevention and treatment window of cardiovascular diseases forward through early detection and early intervention, and estimating the risk of a future CVD event is the first and necessary step [2].

The new non-invasive index of arteriosclerosis, arterial velocity pulse index (AVI) and arterial pressure volume index (API), have been more and more widely used in clinical [3]. AVI represents central arterial pressure and impaired AVI might indicate increased workload on the heart [4]. Furthermore, API reflects reactive vasodilation of peripheral arteries [5]. Arterial stiffness as a predictor of CVD mortality and events, have been less consistent, with some [6, 7] but not others [8] finding an association. However, recent researches did report a significant association between arterial stiffness and CVD outcomes [9].

The Framingham cardiovascular risk score (FCVRS) has contributed to the identification of cardivascular risk factors [10]. Several major studies have found associations between AVI, API value and FCVRS risk score. For the most part, high arterial stiffness is associated with a trend towards increasing CVD risk [11].

However, the association between AVI, API and FCVRS was influenced by many factors, especially age. To date, there is no definite evidence that AVI or API is high in young people, which is related to the high risk of cardiovascular disease in the future. It is necessary to further study and explore the association between AVI, API and CVD risk and its clinical significance.

The present study aimed to evaluate the association between AVI, API value and FCVRS using restrictive cubic spline functions, especially in subjects of different ages, so as to be more accurate prediction of CVD risk.

## 2. Materials and Methods 

### 2.1 Study Population

A total of 4311 volunteers participated in the health care monitoring system at Jiading Branch of Shanghai First People’s Hospital, Shanghai, China. All study protocols were approved by the Ethics Committee of Shanghai General Hospital (approval number: 2019KY009-4) and registered on the official website of China Clinical Trial Registration Center (ChiCTR2000035937), and participants provided written informed consent.

### 2.2 Inclusion and Exclusion Criteria

We included all individuals with arterial stiffness data collected at age 18 years and older and with subsequent follow-up time available. The exclusion criteria were as follows: Subjects who were receiving hemodialysis or had atrial fibrillation; Subjects in whom AVI and API were unable to be obtained due to previous vascular intervention or upper limb amputation or infection; Subjects who were unable to cooperate to complete measurement.

### 2.3 Baseline Measurements

Individual results of a comprehensive health and lifestyle questionnaire for study participants were collected with their consent [12]. Information on age, sex, smoking, alcohol intake, history of hypertension, diabetes and use of medications was obtained by self-administered questionnaire. Smoking was defined as: smoking more than 100 cigarettes in a lifetime [13]. Alcohol intake was defined as: liquor, beer, rice wine, yellow wine or wine was consumed more than once a week on average [14]. Weight and height were measured by nurses following standardized protocols, and body mass index (BMI) calculated as weight/${\mathrm{height}}^{2}$ (kg/$m^{2}$).

### 2.4 Arterial Stiffness Indices and Blood Pressure Measurement

The subjects fully rested for 5~10 min before measurement. Stop smoking and avoid caffein at least 24 hours before the examination. Then a cuff was wrapped around one-side of the upper arm of seated participants. The balloon mark is aligned with the brachial artery, and the lower edge of the cuff is 2~3 cm away from the transverse line of the cubital fossa. The subjects were in the sitting position, and measurements were taken in a quiet, temperature controlled room (24–26 °C). AVI and API data were measured using cuff oscillometry with PASESA AVE-2000Pro (Shisei Datum, Tokyo, Japan) by trained technicians [9, 15]. Systolic blood pressure (SBP), diastolic blood pressure (DBP) and estimated central arterial blood pressure (eCSBP), estimated central artery pulse pressure (eCAPP) using intercepts and coefficients for independent variables were also measured. We calculated eCSBP and eCAPP as follows: eCSBP = 0.1152 × age + 0.7512 × SBP + 0.3095 × DBP + 0.1884 × AVI + 0.4001 × API – 0.1105, and eCAPP = 0.1496 × age + 0.1088 × SBP + 0.7312 × PP + 0.2163 × AVI + 0.3649 × API – 12.3859 [16]. Rest for 2 min and measure again. Take the average value of the two times as the final result.

### 2.5 Framingham Cardiovascular Risk Score

FCVRS was calculated to estimate 10-year cardiovascular risk using the following equation [17], including gender, age, smoking, total cholesterol (TC), high-density lipoprotein cholesterol (HDL-C) and SBP. The FCVRS was calculated for each patient using the National Cholesterol Education Program (NCEP) risk score calculator [18].

### 2.6 Other Variables

Hypertension was defined as SBP at least 140 mmHg and/or DBP at least 90 mmHg, and/or antihypertensive drug used [19]. Diabetes mellitus is defined as a glycosylated hemoglobin (Hb) at least 6.5% and/or fasting glucose at least 7 mmol/L and/or the use of oral hypoglycemic agents or insulin therapy [20]. Dyslipidemia was defined as total cholesterol more than 6.61 mmol/L and/or triglycerides more than 1.7 mmol/L after an overnight fast and/or the presence of lipid lowering therapy.

### 2.7 Blood Biochemical Analysis

The subjects should be fasting for more than 12 hours, and 5 mL of venous blood was drawn the next morning. The relevant indexes were measured by immunoturbidimetry with automatic biochemical instrument, including TC, HDL-C, low-density lipoprotein cholesterol (LDL-C), triglyceride (TG), glucose levels, etc.

### 2.8 Statistical Analysis

According to the age division of the World Health Organization, the age of subjects is divided into three levels: 18~44 years, 45~59 years, ≥60 years. The data were presented as numbers and percentages by category for qualitative variables and as mean and standard deviation, or as median and interquartile range if the distribution did not appear to follow a normal distribution, for quantitative variables. SPSS 19.0 (IBM, Armonk, NY, USA) statistical analysis software was used. The continuous data were compared using one way analysis of variance for inter group comparison, and Q-test was used for intra group comparison. The categorical variables between groups were compared by chi square test. The relations of AVI and API to all other variables were analyzed using a multivariate linear regression analysis and Pearson’s correlation coefficient. The stepwise multiple linear regression analyses was used to evaluate the independent associations between AVI or API and clinically relevant variables. The following variables were included in the analysis: clinical data (age, sex, BMI, HR, SBP, DBP, TC, glucose), current smoking, alcohol consumption, history of hypertension, diabetes and dyslipidemia, medications of antihypertension and antidiabetes. The kicking boundary value was α_enter_ = 0.05, α_out_ = 0.10 [21]. The relationship between FCVRS and AVI, API were analyzed by restrictive cubic spline (RCS). We selected 5 knots for data, which are 5th, 27.5th, 50th, 77.5th and 95th. RCS statistical analyses were performed using Stata 12 (Stata Corp, College, Station, TX, USA). *p *< 0.05 represented that the difference was statistically significant.

## 3. Results

### 3.1 Baseline Characteristics

A total of 4311 participants were included in this study. The characteristics of all participants are shown in Table 1. The average age of participants was 58 years, and 48.5% were male. According to age, 755 participants (17.51%) aged 18~44 years, 1260 participants (29.23%) aged 45~59 years, and 2296 participants (53.26%) aged 60 and over. The mean (± SD) AVI, API and FCVRS were 17.90 ± 6.38, 29.36 ± 7.21 and 11.04 ± 5.99, respectively.

**Table 1.** **Comparison of basic characteristics of 4311 participants**.

Item	18–44 years age	45–59 years age	≥60 years age	F/$\chi^{2}$	*p* value
	(n = 755)	(n = 1260)	(n = 2296)		
Sex (Men)	355 (47.0%)	538 (42.7%)	1198 (52.2%)	30.07	<0.001
BMI (kg/$m^{2}$)	24.64 ± 4.39	24.74 ± 3.38	24.04 ± 3.35*#	18.73	<0.001
Current smoker (n)	32 (4.2%)	63 (5.0%)	163 (7.1%)	11.33	0.003
Alcohol consumption (n)	16 (2.1%)	28 (2.2%)	77 (3.4%)	5.40	0.067
Hypertension (n)	179 (23.7%)	470 (37.3%)	1073 (46.7%)	130.75	<0.001
Diabetes mellitus (n)	75 (9.9%)	253 (20.1%)	708 (30.8%)	151.21	<0.001
Dyslipidemia (n)	210 (27.8%)	400 (31.7%)	631 (27.5%)	7.64	0.022
Medications					
Antihypertension (n)	86 (11.4%)	356 (28.4%)	1021 (44.5%)	302.93	<0.001
Antidiabetes (n)	46 (6.1%)	176 (14.0%)	509 (22.2%)	115.57	<0.001
Systolic blood pressure (mmHg)	121.88 ± 20.34	130.74 ± 21.95*	137.49 ± 23.50*#	143.78	<0.001
Diastolic blood pressure (mmHg)	79.49 ± 13.72	82.17 ± 13.39*	79.01 ± 13.04#	23.70	<0.001
Heart rate (beats/min)	83.52 ± 12.87	79.26 ± 12.52*	78.74 ± 12.46*	42.53	<0.001
Total cholesterol (mmol/L)	4.40 ± 0.93	4.61 ± 1.01*	4.42 ± 1.06#	16.09	<0.001
Triglyceride (mmol/L)	1.48 ± 1.17	1.58 ± 1.13	1.46 ± 0.90#	5.18	0.006
HDL cholesterol (mmol/L)	1.10 ± 0.28	1.12 ± 0.31	1.12 ± 0.33	1.93	0.145
LDL cholesterol (mmol/L)	2.83 ± 0.90	2.93 ± 0.93*	2.75 ± 0.99#	16.10	<0.001
Glucose (mmol/L)	5.34 ± 1.69	5.56 ± 1.09	6.04 ± 1.60	2.03	0.136
eGFR (mL/min)	134.18 ± 37.60	125.69 ± 43.32*	110.15 ± 38.56*#	104.30	<0.001
AVI	12.92 ± 3.99	17.48 ± 5.67*	19.78 ± 6.47*#	391.99	<0.001
API	25.68 ± 5.61	27.61 ± 6.32*	31.53 ± 7.38*#	269.84	<0.001
eCSBP (mmHg)	118.10 ± 18.05	124.25 ± 18.70*	129.39 ± 19.69*#	105.70	<0.001
eCAPP (mmHg)	35.85 ± 10.53	41.39 ± 11.31*	48.74 ± 13.78*#	347.54	<0.001
FCVRS	0.59 ± 4.57	10.43 ± 2.86*	14.81 ± 2.38*#	6383.86	<0.001

Data are shown as mean ± SD, percent or number (%) of event outcomes. BMI, body mass index; eGFR, estimated glomerular filtration rate; AVI, arterial velocity pulse index; API, arterial pressure volume index; eCSBP, estimated central arterial blood pressure; eCAPP, estimated central artery pulse pressure; SD, standard deviation. Compared with the group 18–44 years old, *p < 0.05; Compared with the group 45–59 years old, #*p *< 0.05.

### 3.2 Relations between AVI, API and Clinical Variables

**Fig. 1.**
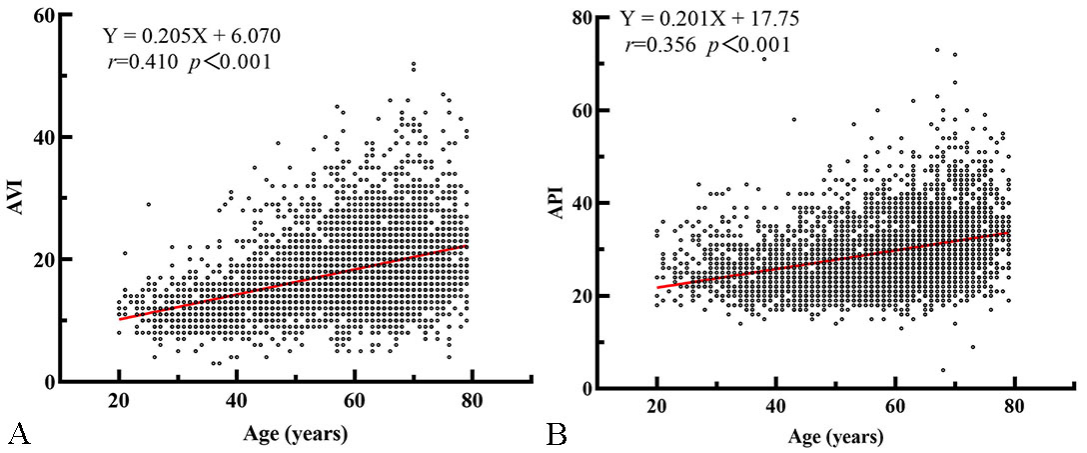
**Scatter plot and linear regression curve of AVI and API with age**. (A) There is a significant positive correlation between age and AVI. (B) There is a significant positive correlation between age and API.

In the full cohort, both AVI and API were associated with several variables. Table 2 shows correlation coefficients between AVI or API and variables. Particularly, both AVI and API were strongly correlated with age (*r* = 0.410, 0.356, *p *< 0.001) and SBP (*r* = 0.385, 0.691, *p *< 0.001). Scatter plots of API and AVI with age are shown in Fig. 1.

**Table 2.** **Coefficients of correlation between API or AVI and other variables**.

Item	AVI		API	
	r	*p* value	r	*p* value
Age	0.410	<0.001	0.356	<0.001
Sex	–0.010	0.531	–0.008	0.620
BMI (kg/$m^{2}$)	–0.042	0.006	0.144	<0.001
SBP (mmHg)	0.385	<0.001	0.691	<0.001
DBP (mmHg)	0.124	<0.001	0.066	<0.001
HR (beats/min)	–0.209	<0.001	–0.098	<0.001
Total cholesterol (mmol/L)	0.004	0.795	–0.024	0.119
Triglyceride (mmol/L)	0.004	0.773	0.050	0.001
HDL cholesterol (mmol/L)	0.039	0.011	–0.047	0.002
LDL cholesterol (mmol/L)	–0.030	0.047	–0.033	0.029
Glucose (mmol/L)	0.063	<0.001	0.126	<0.001

Table 3 shows the results of multiple linear regression analysis of AVI or API using the stepwise method. After adjustments for confounding factors, age, sex, BMI, SBP, HR, history of hypertension were all independently associated with AVI and API. DBP, TC, history of diabetes mellitus were independently associated with API.

**Table 3.** **Multiple regression analysis of API or AVI (stepwise)**.

	AVI				API			
	β	S.E	*p* value	VIF	β	S.E	*p* value	VIF
Age	0.142	0.007	<0.001	1.138	0.031	0.005	<0.001	1.240
Sex	–0.811	0.165	<0.001	1.024	–0.451	0.128	<0.001	1.101
BMI (kg/$m^{2}$)	–0.161	0.024	<0.001	1.126	0.145	0.018	<0.001	1.125
SBP (mmHg)	0.108	0.005	<0.001	2.351	0.289	0.004	<0.001	2.714
HR (beats/min)	–0.097	0.007	<0.001	1.039	–0.028	0.005	<0.001	1.076
Hypertension	–0.766	0.248	0.002	2.226	1.368	0.191	<0.001	2.355
Dyslipidemia	–0.367	0.184	0.047	1.049		Removing		
DBP (mmHg)		Removing			–0.300	0.007	<0.001	2.018
Total cholesterol (mmol/L)		Removing			–0.149	0.060	0.014	1.033
Diabetes mellitus		Removing			0.354	0.148	0.017	1.072

β is the regression coefficient; S.E is the standard error; VIF is the valiance inflation factor.

### 3.3 Correlations of API and AVI with FCVRS

**Fig. 2.**
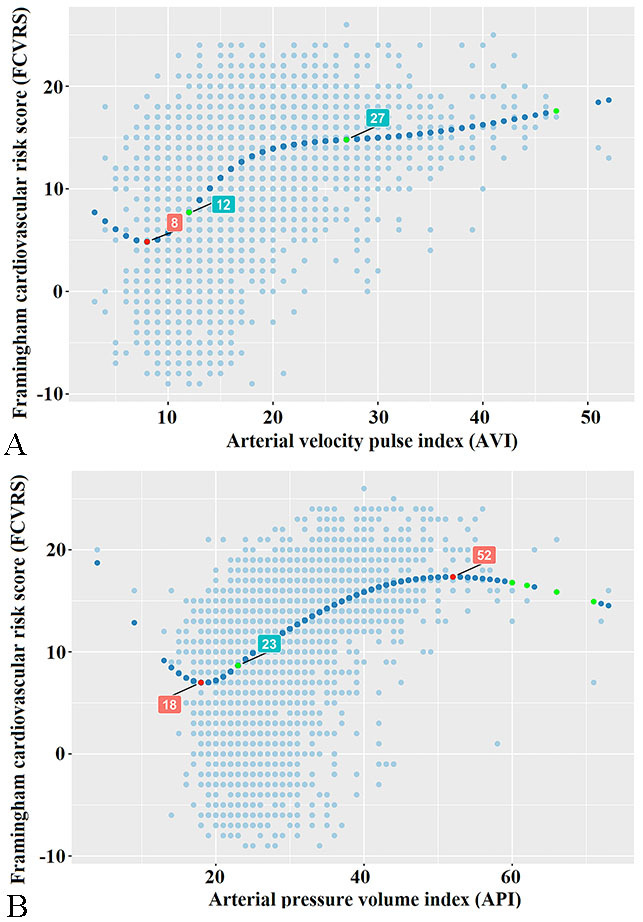
**Correlations of API and AVI with FCVRS based on Restricted Cubic Spline Functions**. (A) AVI and FCVRS have a significant U-shaped dose-response relationship. The AVI value associated with the lowest risk score of cardiovascular disease is 8 units. (B) There is a significant U-shaped dose-response relationship between API and FCVRS, and 18 units of API values are associated with the lowest risk score for cardiovascular disease.

Table 4 shows correlation coefficients between FCVRS and previous arterial stiffness indices, and statistically associations were found for all indices. However, although the relationships were significant, they were not strong. In order to further explore the relationship between AVI or API and FCVRS, a restrictive cubic spline was used, the curves were drawn. The results showed that: AVI or API and FCVRS showed a significant U-type dose-response relationship. The AVI value associated with the lowest risk score of cardiovascular disease was 8 units, and the API value associated with the lowest risk score of cardiovascular disease was 18 units. After AVI increased to 12 units, the risk of FCVRS increased rapidly afterwards until abount 27 units, the increasing trend of FCVRS was relatively flat. In addition, when API was in the range of 0–18 units, the increasing of FCVRS showed a downward trend. After API increased to 23 units, FCVRS was increasing rapidly. After API increased to 52 units, FCVRS showed a relatively stable increasing trend (Fig. 2).

**Table 4.** **Correlation coefficients between FCVRS and arterial stiffness indices**.

Item	FCVRS	
	r	*p* value
AVI	0.435	<0.001
API	0.417	<0.001
eCSBP	0.342	<0.001
eCAPP	0.464	<0.001

## 4. Discussion

The study was to evaluate the relationships of the API or AVI with cardiovascular risk as defined by the FCVRS. We observed that AVI or API and FCVRS have a significant U-shaped dose-response relationship. AVI value associated with the lowest risk score of cardiovascular disease was 8 units. After AVI increased to 12 units, the risk of FCVRS increased rapidly afterwards. While, after AVI increased to 27 units, the increasing trend of FCVRS was relatively flat. In similarly, API value associated with the lowest risk score of cardiovascular disease was 18 units. After API increased to 23 units, the risk of FCVRS increased rapidly. While, After API increased to 52 units, FCVRS showed a relatively stable increasing trend. API value associated with the starting point of FCVRS increase flat period was 52 units.

AVI and API, as a non-invasive indices of artery stiffness, is an important surrogate marker of vascular damage. These indices may have variability among investigators, however, the evaluation of AVI or API is convenient and effective, and has been widely used in clinical [9, 22]. In recent years, reports have indicated that AVI or API, was strongly associated with the occurrence of cardiovascular events and Framingham cardiovascular disease risk [9, 23, 24].

Our results are consistent with previous studies showing that AVI and API were associated with typical risk factors for arterial stiffness such as age, sex, BMI and diseases (hypertension, dyslipidemia, diabetes) [25]. We also observed a linear correlation between AVI or API and FCVRS, but the correlation is moderate. To the best of our knowledge, no study has been carried out to evaluate non-linear association between the API or AVI and FCVRS in a large population. However, our results suggest important non-linearity in the association between AVI or API and FCVRS.

The association between AVI, API and FCVRS may be driven by confounders, such as age, blood pressure and BMI [24]. However, blood pressure increases with age [26], arterial stiffness is strongly associated with aging [27], and age is also one of the important factors in FCVRS [28]. There was a strong interactions with age in the associations between AVI or API and FCVRS, meaning that the non-linearity association might be affected by age.

In our study, AVI or API value associated with the lowest risk score of cardiovascular disease was 8 or 18 units. According to the linear analysis of AVI, API and age, these subjects with the lowest risk score of cardiovascular disease mainly in the youth group. In addition, AVI or API value associated with the starting point of flat period of cardiovascular disease risk was 27 or 52 units, and these subjects mainly in the elderly group. Studies in arterial stiffness have shown that the proportion and structure of elastin and collagen in arterial wall changed with the increase of age, vascular elasticity decreased and the artery stiffness increased [29, 30]. In addition, there may be related to the development of vascular endothelial function. Bhangoo *et al*. [31] reported that endothelial peripheral arterial tonometry index, a measure of small artery endothelial function, increased with pubertal progression and was significantly correlated with age in healthy children and adolescents. As this study showed, API and AVI are both BP-dependent indices, and the mechanism of their influence on the U-shaped relationship between AVI, API and FCVRS deserve further investigation.

## 5. Limitations

This study has several limitations. Firstly, the medical treatments for hypertension or diabetes at baseline may influenced the study results of AVI and API, we did not do a subgroup study based on the classification of disease. In the future, subgroup adjustment could be made for these factors as well as other potential biochemical confounders. Second, ultrasonic echocardiography, coronary angiography were not performed, and we did not observe the end event, the effect of AVI, API has on the development of cardiovascular diseases are required to be further studied in the future.

## 6. Conclusions

In conclusion, API or AVI had a U-shaped association with FCVRS, and the AVI associated with the lowest risk score of cardiovascular disease was 8 units, the API associated with the lowest risk score of cardiovascular disease was 18 units. An improved understanding of the associations between AVI or API and FCVRS may provide a new perspective for early treatment or lifestyle modifications to prevent CVD.
